# Microbial influencers: the airway microbiome’s role in asthma

**DOI:** 10.1172/JCI184316

**Published:** 2025-02-17

**Authors:** Young Jin Kim, Supinda Bunyavanich

**Affiliations:** 1 Division of Allergy and Immunology, Department of Pediatrics, and; 2 Department of Genetics and Genomic Sciences, Icahn School of Medicine at Mount Sinai, New York, New York, USA.

## Abstract

Asthma is a common chronic respiratory disease affecting people of all ages globally. The airway hosts diverse microbial communities increasingly recognized as influential in the development and disease course of asthma. Here, we review recent findings on the airway microbiome in asthma. As relationships between the airway microbiome and respiratory health take root early in life, we first provide an overview of the early-life airway microbiome and asthma development, where multiple cohort studies have identified bacterial genera in the infant airway associated with risk of future wheeze and asthma. We then address current understandings of interactions between environmental factors, the airway microbiome, and asthma, including the effects of rural/urban environments, pet ownership, smoking, viral illness, and antibiotics. Next, we delve into what has been observed about the airway microbiome and asthma phenotypes and endotypes, as airway microbiota have been associated with asthma control, severity, obesity-related asthma, and treatment effects as well as with type 2 high, type 2 low, and more newly described multi-omic asthma endotypes. We then discuss emerging approaches to shape the microbiome for asthma therapy and conclude the Review with perspectives on future research directions.

## Introduction

Asthma affects millions of people of all ages and walks of life worldwide and exerts extensive effects on global health. Airway inflammation in asthma leads to airflow obstruction and bronchial hyperresponsiveness, which are experienced by the individual as shortness of breath, cough, and/or wheezing that can be unpredictable and severe for some (1). Shaped by complex interactions between intrinsic and extrinsic factors, asthma is highly diverse in its presentation and disease course, posing challenges for its diagnosis and management.

**Figure 1:**
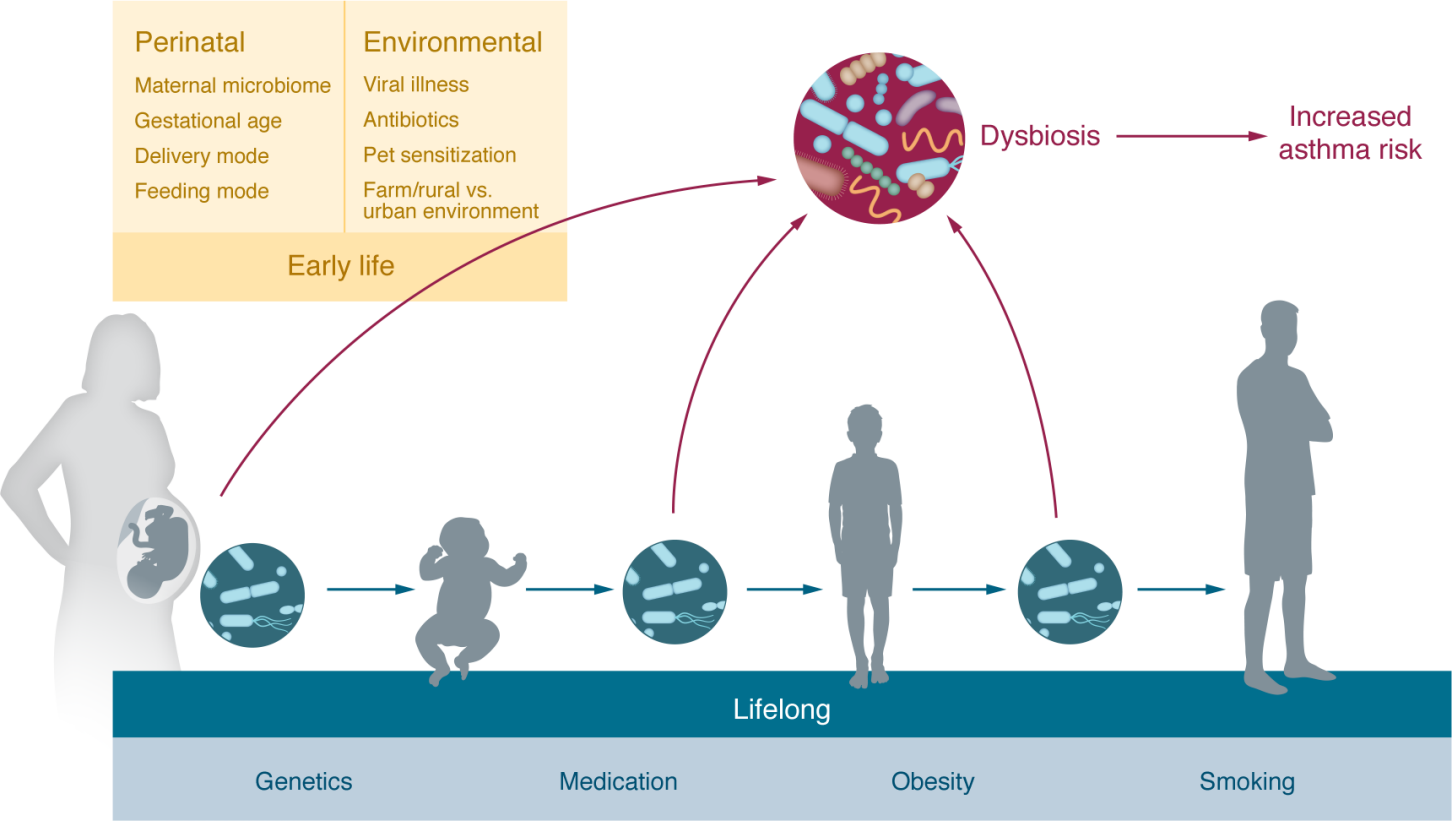
Many factors influence the airway microbiome and asthma early and throughout life. Perinatal factors and early-life environmental exposures affect the development of the airway microbiome. Growing up in farm and rural environments has been found to be protective against asthma, while smoking, respiratory infections, and antibiotic use increase asthma risk. All of these environmental factors have been associated with airway dysbiosis. Additional factors, including genetics, obesity, medication use, and smoking, can influence the airway microbiome throughout life.

Many lines of evidence support microbial influences on the pathogenesis and trajectories of asthma, motivating investigations of the microbiome in asthma (Figure 1). High-throughput sequencing technologies, including 16S rRNA and metagenomic sequencing to systematically profile the microbiome in culture-independent manners, has and will continue to revolutionize our understanding of the microbiome in health and disease (2). Methodical study of the microbiome of varying anatomic locations of the human body has demonstrated that the structure, function, and diversity of the microbiome in different anatomic niches differs widely (3). While much research has focused on relationships between the gut microbiome and asthma, this Review specifically focuses on the airway microbiome in asthma, the niche most proximal to this prevalent chronic respiratory condition.

**Figure 2:**
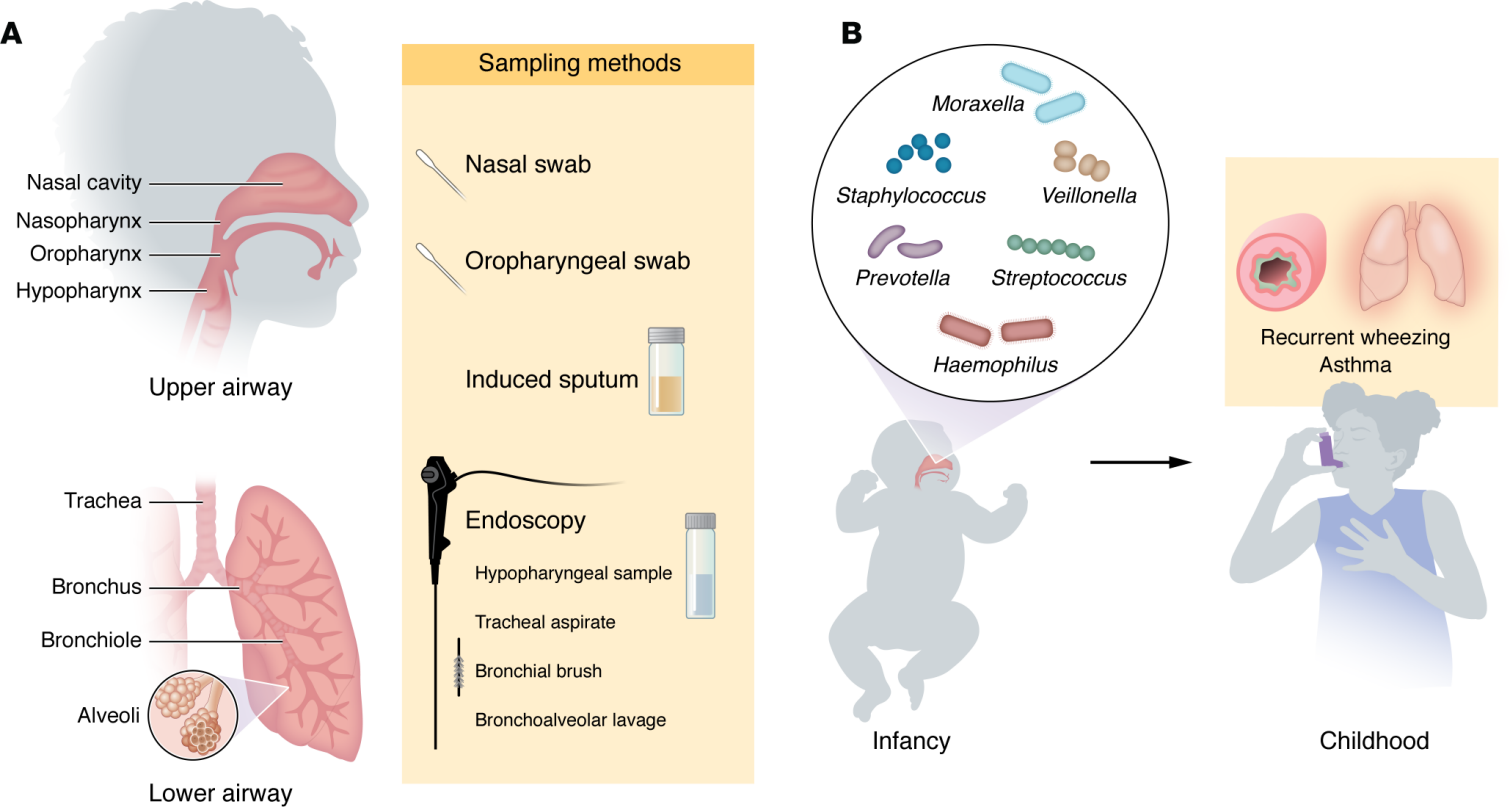
Airway sampling for microbiome studies and bacterial genera in the infant upper airway microbiome that have been associated with childhood asthma outcomes. (**A**) Upper and lower airway sampling can be undertaken by multiple approaches. Swabs can be used to obtain samples from the nasal passages, nasopharynx, and oropharynx. Endoscopy or sputum induction can be performed to obtain samples from the hypopharynx and lower airway. (**B**) Studies of upper airway samples collected from infants enrolled in longitudinal cohort studies have identified upper airway genera associated with the development of childhood wheeze and asthma.

Even along the airway, there is a microbial biogeography where microbial communities vary by specific location and sampling approach (4). Reflecting the expanse of locations that comprise the airway, studies of the airway microbiome in asthma have relied on specimens collected from several locations along the upper and lower airway (Figure 2A). This diversity of sample types across studies has contributed to a very wide array of reported findings, with this heterogeneity of observations further compounded by differences in the populations studied, study designs, and analytic methods (Table 1). Thus, while there are some shared findings across the investigations reviewed in this article, there are also many observations that contrast.

As relationships between the airway microbiome and respiratory health take root early in life, we begin this Review with an on overview of the early-life airway microbiome and asthma development (Figure 1). We address current understandings of interactions among environmental factors, the airway microbiome, and asthma. Next, we delve into what has been observed about the airway microbiome associated with specific asthma phenotypes and endotypes. We then discuss approaches to shape the microbiome for asthma therapy and conclude the Review with perspectives on future research directions.

## The early-life airway microbiome and asthma development

The airway microbiome undergoes dynamic changes throughout life in both health and disease. Longitudinal studies in multiple cohorts have collectively identified that the relative abundances of many genera in the airway microbiome during infancy are associated with childhood asthma outcomes, including future wheeze and future asthma (Figure 2B).

Most of what we know about the relationships between the early-life airway microbiome and asthma development is based on studying upper airway samples collected from infants enrolled in longitudinal studies where they are followed over time for the development of wheeze and asthma. Bronchoscopy for lower airway profiling is more invasive than upper airway sampling. Healthy children do not undergo bronchoscopy, and the procedure is not commonly done in children with asthma. However, one study jointly profiled and directly compared the upper and lower airway microbiomes of 27 US children with severe asthma, each of whom underwent bronchoscopy with dual sampling of the nasal and bronchial airways (5). Systematic analyses revealed significant compositional differences between the nasal and bronchial airways, with *Corynebacterium, Staphylococcus,* and *Moraxella* most prevalent in the nasal airway, and *Veillonella, Prevotella, Streptococcus,* and *Neisseria* most common in the bronchial airway (5). The nasal microbiome formed a dense network with *Moraxella* and *Alloiococcus* as hubs, whereas the bronchial network had 1.6-fold less connectivity per genus and no clear hubs (5). In terms of interactions between the upper and lower airways in severe asthma, bronchial *Porphyromonas* had the highest number of associations with nasal genera (11% of the total number of associations), and nasal *Corynebacterium* had the highest number of associations with bronchial genera (8% of the total number of associations) (5).

With respect to the relationship between the infant upper airway microbiome and the development of asthma, a landmark study of hypopharyngeal samples from 321 neonates in the Copenhagen-based longitudinal study COPSAC2000 found using culture-based methods that upper airway colonization at 1 month of age with *Streptococcus pneumoniae*, *Haemophilus influenzae*, and *Moraxella catarrhalis* was associated with future wheeze, asthma at 5 years of age, and blood eosinophil count and total but not specific IgE at 4 years of age (6). Subsequent studies employing 16s rRNA sequencing have provided corroborating as well as distinct findings. Consistent with the Danish findings, an Australian cohort study of nasopharyngeal samples from 244 children that employed 16S rRNA sequencing found that the frequency of illness-associated microbiome profile groups dominated by *Streptococcus*, *Haemophilus*, or *Moraxella* during the first 2 years of life was associated with chronic wheeze at 5 years of age (7). A population-based birth cohort study of 704 children in Finland provided further insight on early-life *Moraxella* in the upper airway, finding that persistent *Moraxella* sparsity in nasal samples during ages 2–13 months was associated with higher risk of asthma at 7 years of age (8).

Other studies have identified additional upper airway microbes associated with asthma development. A subsequent study by the Copenhagen Prospective Studies on Asthma in Childhood (COPSAC) group, but of a different cohort of 700 Danish children (COPSAC2010), employed 16S rRNA sequencing to assess the microbiome of hypopharyngeal aspirates at ages 1 week, 1 month, and 3 months and their association with asthma by 6 years of age (9). Here, they found that microbial diversity and the relative abundances of *Veillonella* and *Prevotella* at 1 month of age were associated with asthma by age 6 years (9). The contrast between these findings and those from the earlier COPSAC2000 cohort was attributed to the newer cohort’s use of 16S rRNA sequencing rather than culture (9). Researchers also employing 16S rRNA sequencing but studying an independent population-based birth cohort of 159 children from London, United Kingdom, for the development of physician-diagnosed wheeze by the age of 24 months found a different direction of effect for *Prevotella*, observing that decreasing *Prevotella* abundances in oropharyngeal swabs were associated with wheeze (10). Additionally, these investigators found that increases in *Neisseria* and decreases in *Granulicatella* were associated with wheeze (10). While there was no association between hypopharyngeal or oropharyngeal *Staphylococcus* spp. and wheeze or asthma in the earlier studies, investigators of the Wisconsin, US–based Childhood Origins of Asthma (COAST) cohort of 285 children found that a nasopharyngeal mucus microbiome trajectory during the first 6 months of life dominated by *Staphylococcus* was associated with the number of wheezing illnesses by the age of 3 years and physician-diagnosed asthma from 6–18 years of age (11). Distinct from those findings and more recently, a study of the nasal microbiota of urban children from four centers across the United States at age 12 months (*n* = 120) and 36 months (*n* = 142) found that while there was no significant relationship between 12-month nasal microbiota composition and longitudinal respiratory phenotype defined by different levels of wheezing (low, transient, high) and atopy (low, high), nasal microbiota dominated by *Moraxella* spp. were associated with atopy regardless of high or low wheeze (12).

Together, these studies found that the presence of a number of bacterial genera in the early-life upper airway were risk factors for the development of future wheeze and asthma (Figure 2B). Reducing the abundances of these microbes in the early-life airway could represent a novel strategy for asthma prevention.

## Environmental factors, the airway microbiome, and asthma

Host and environmental factors can shape the airway microbiome (13–16). Both the airway microbiome and asthma are influenced by a multitude of environmental exposures, including home and school environment, infections, medication use, and smoking (Figure 1).

### Farm versus urban environment.

Farm environment exposure, particularly in early life, has been associated with a protective effect against asthma in multiple cohorts. A study of environmental exposures and immune profiles of children from agricultural populations compared 30 Amish children (whose communities follow traditional farming practices) and 30 Hutterite children (whose communities use industrialized farming practices) and found significantly lower asthma prevalence in Amish children (0% vs. 20%), with Amish home dust containing 6.8 times higher endotoxin levels (17). Dust from asthma-protective farms was also found to contain animal and plant proteins, including fatty acid metabolites that might regulate airway responses (18). Conversely, urban environments are associated with increased asthma risk. Within the COPSAC2010 cohort of 700 Danish children, infants in urban environments had higher odds of developing asthma (adjusted OR = 2.31, 95% CI = 1.47–3.68) and aeroallergen sensitization (adjusted OR = 1.77, 95% CI = 1.05–3.02) by 6 years of age compared with those living in rural environments (19). The microbiome of hypopharyngeal aspirates collected at age 1 week, 1 month, and 3 months was more homogenous than aspirates from rural children at the same time points, and urban living was consistently associated with the relative abundance of *Veillonella* in hypopharyngeal aspirates at all time points (19). The degree to which these differential airway microbiota play an active versus bystander role in the complex relationships between urban versus rural environmental exposures and asthma risk remains to be seen.

### Pet ownership.

Many studies of pet ownership and asthma risk in the United States have shown inverse relationships (20–24), but the results have not been consistent globally, with a recent meta-analysis of 77,000 children from the European Union Child Cohort Network concluding that early-life cat and dog ownership do not increase the risk of school-age asthma, but they may increase risks of pet sensitization (25). A study of 132 school-age children from the New York metropolitan area with persistent asthma examined the nasal microbiome associated with cat and dog allergen sensitization (26). Pet sensitization was prevalent in this sample, with 68.9% sensitized to cat and 72.7% sensitized to dog. The relative abundances of *Corynebacterium* sp. and *Staphylococcus epidermidis* were associated with reduced pet sensitization, and causal mediation analysis showed that these protective effects were mediated by reduced nasal expression of genes related to IgE-mediated sensitization and mast cell function, including *TPSAB1*, *GATA2*, and *CPA3* (26). These results suggest that the effect of pet sensitization on asthma risk may be partly mediated by the modulation of host immunity system by airway microbiota.

### Smoking.

Smoking, a global influence on respiratory health, affects lung function and the airway microbiome. Among a general population sample of 529 Australian adults, smoking was associated with diversity loss, disruption of microbial networks, and expansion of *Streptococcus spp*. in posterior oropharyngeal swabs (27). A study of tracheal aspirates from 298 intubated children from the United States similarly found diversity loss associated with cigarette smoke exposure in addition to higher relative abundances of *Serratia app., Moraxella spp., Haemophilus spp.*, and *Staphylococcus aureus* (28).

Additional studies have examined the effects of smoking on the airway microbiome of individuals with asthma. The European Unbiased Biomarkers for the Prediction of Respiratory Disease Outcomes (U-BIOPRED) cohort study comparing sputum metagenomics of adults with severe asthma with (*n* = 33) and without (*n* = 64) smoking history revealed that ever smokers had more *Veillonella* spp. and *Prevotella* spp. and less *Moraxella catarrhalis* and *Neisseria sp*. compared with never smokers (29). Shannon diversity, a measure of α-diversity (Table 1), among individuals with severe asthma did not significantly differ based on smoking status (29). These smoking-associated changes in the airway microbiome may contribute to the increased risk and severity of asthma observed in smokers and those exposed to secondhand smoke.

### Respiratory infections.

Respiratory infections in early life, particularly those caused by respiratory syncytial virus (RSV) and rhinoviruses (RV), alter the airway microbiome and influence future asthma risk (30). Our understanding of the effects of illness on the airway microbiome and asthma are largely based on upper airway samples given the relative ease of sampling the upper versus lower airway (Figure 2A) overall and especially during acute illness when asthma is often exacerbated and bronchoscopy is avoided.

A cohort study of 112 Dutch infants followed from birth with several nasopharyngeal sampling time points found that children experiencing more than 2 respiratory infections during infancy demonstrated aberrant microbial development of their nasopharyngeal microbiome, including reduction of *Corynebacterium* and *Dolosigranulum*, early enrichment with *Moraxella*, and later enrichment with *Neisseria* and *Prevotella* spp. (31). Among 842 infants hospitalized for bronchiolitis across 17 US centers in the Multicenter Airway Research Collaboration (MARC-35) cohort, researchers observed enrichment of *Moraxella* or *Streptococcus* spp. in nasal samples that was associated with recurrent wheezing by the age of 3 years (32), supporting earlier findings from an Australian prospective cohort of 234 children that incursions of *Streptococcus, Moraxella*, and *Haemophilus* mark virus-associated respiratory infections (33). Within a subset of the MARC-35 cohort (*n =* 122), additional analyses examining the nasopharyngeal microbiome with other data identified RV bronchiolitis endotypes associated with higher risk of recurrent wheeze and asthma, including endotypes marked by RV-C infection, *Moraxella*-dominant microbiota, and high T2 cytokine response (34). The investigators also described endotypes of RSV bronchiolitis among 221 infants in MARC-35 using a similar approach, including an endotype of *S*. *pneumoniae* and *M*. *catarrhalis* codominance and high IFN-α and -γ responses that was associated with higher odds of asthma compared with a reference endotype (35). Additionally, the investigators profiled nasopharyngeal metatranscriptomics in 244 MARC-35 children to describe profiles marked by different combinations of RSV, RV types, and nasopharyngeal bacteria, including commensals, *H*. *influenzae, S*. *pneumoniae, M*. *nonliquefaciens*, and *M*. *catarrhalis*, associated with different rates of eczema, asthma, cytokine expression, antibiotic resistance, and lipid metabolism of the microbiome (36).

Differences in the airway microbiome with COVID-19 infection are observed, yet it remains unclear if these differences led to COVID susceptibility or if COVID-19 infection led to changes in the airway microbiome (37). One small cross-sectional study of nasopharyngeal samples from 48 adult US patients with COVID-19 found no community-level differences between patients with asthma (*n* = 7) and those without (*n* = 41), although genus-level analyses showed decreased abundances of *Porphyromonas, Haemophilus, Moraxella*, and *Campylobacter* in individuals with asthma versus those without (38). Given limited data on this topic, additional studies are needed to characterize potential relationships among the airway microbiome, COVID-19, and asthma.

Overall, there is a substantial body of evidence supporting associations between respiratory infections from a variety of pathogens and changes to the airway microbiome, but it remains tricky to disentangle directional relationships between them. Understanding these causal relationships could reveal insights on the role that airway microbiota might play in virus-induced asthma pathogenesis and exacerbations.

### Antibiotic exposure.

A substantial volume of research supports that infant antibiotic use is associated with the development of childhood asthma, but evidence that changes in the human microbiome cause this effect is limited (39). While gut microbiota may mediate relationships between antibiotic exposure and asthma risk (40), few studies have examined causal relationships among antibiotic use, airway microbiota, and asthma onset. A population-based Finnish birth cohort study of 697 children studied systemic antibiotic use during 0–11 months of age, physician-diagnosed asthma by the age of 7 years, and nasal microbiome at 2, 13, and 24 months of age (41). Causal mediation analysis revealed that antibiotic use in the first 11 months of life was associated with a 4.0% (95% CI, 0.9%–7.2%) increase in asthma risk (41). 16% of this effect was mediated by longitudinal changes in nasal microbiota (41). Murine model studies have sought to shed light on potential mechanisms linking antibiotic use, microbiome changes, and asthma development. A recent study found that mice given antibiotics in early life were more susceptible to house dust mite–induced allergic airway inflammation associated with short-term disruption of the gut microbiome and decreased systemic levels of indole-2-propionic acid that reduced mitochondrial respiration and altered cytokine production in the respiratory epithelium (42). Notably, antibiotics did not significantly alter lung microbiota in these mice (42). Further studies in large, longitudinally followed cohorts with robust accounting for potential confounders are needed to more fully understand relationships among infant antibiotic use, the respiratory microbiome, and asthma development.

## The airway microbiome of asthma phenotypes and endotypes

**Figure 3:**
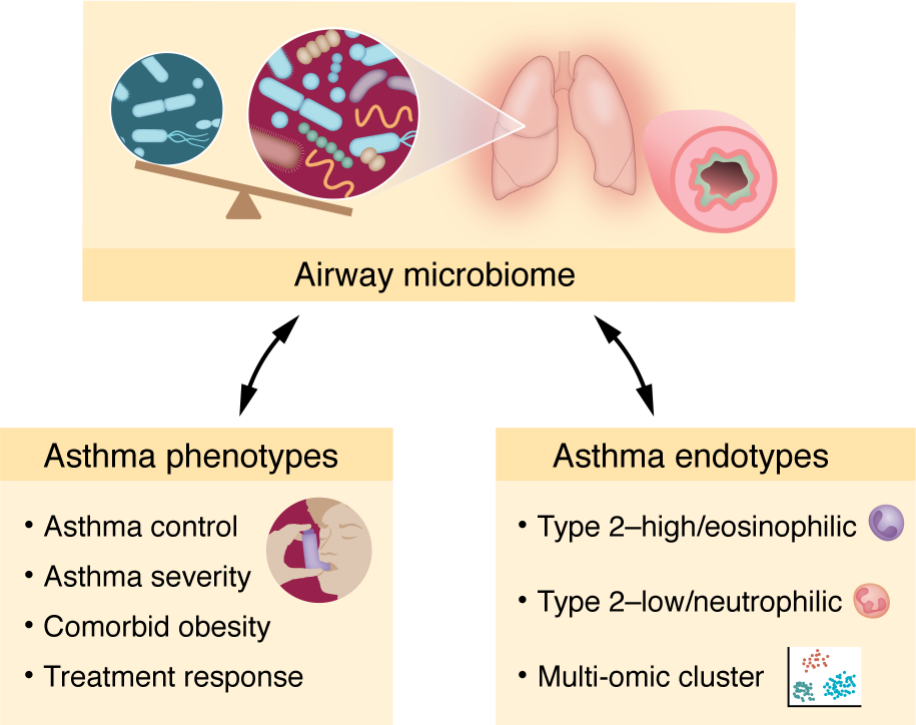
Phenotypes and endotypes of asthma are associated with distinct airway microbiota. Differences in airway communities have been associated with asthma phenotypes, including subtypes based on asthma control, severity, obesity, and treatment response. Airway microbiome characteristics have also been associated with asthma endotypes defined based on prevalent cellular type, predominant immunologic pathway, or multi-omic cluster.

Given the highly variable presentation and disease course of asthma, many efforts have gone into subtyping asthma into phenotypes and endotypes (43). Asthma phenotypes include subtypes based on asthma control, severity, obesity, and treatment response, for example, while various asthma endotypes have been defined based on prevalent cellular type (e.g., eosinophilic, neutrophilic), predominant immunologic pathway (e.g., type 2), or multi-omic cluster (43) (Figure 3). Accordingly, investigators have examined the airway microbiome associated with varying phenotypes and endotypes of established asthma.

### Asthma control.

Optimal control of asthma to reduce or eliminate symptoms is a centerpiece of asthma care (44). To address the hypothesis that different communities of airway microbiota are associated with asthma control, investigators studied the nasal microbiome in 72 adult participants from the United States, including those with exacerbated asthma, nonexacerbated asthma, and individuals acting as healthy controls, finding significantly different community compositions between the groups. Relative to controls, the nasal microbiota of participants with asthma were enriched with taxa from *Bacteroidetes* (45). Four species (*Prevotella buccalis, Dialister invisus, Gardnerella vaginalis*, and *Alkanindiges hongkongensis*) distinguished the participants with exacerbated asthma, nonexacerbated asthma, and individuals acting as healthy controls, with significant enrichment of *Prevotella, Alkanindiges*, and *Gardnerella* in the nasal microbiome of participants with exacerbated asthma (45). Quantitative PCR confirmed the observed species overrepresentation, and metagenomic inference revealed differences in glycerolipid metabolism (45). In a subsequent study of 250 European individuals aged 8–85 years with asthma treated with inhaled corticosteroids (ICS), investigators found that those with exacerbations have lower nasal bacterial diversity and richness compared with those without exacerbations (46). As in the earlier study of US adults, these investigators also found nasal *Prevotella* and *Dialister* to be associated with exacerbations despite ICS treatment, in addition to multiple other nasal and pharyngeal genera (46).

Findings on airway microbiota associated with asthma exacerbations in children have differed from those of adults. Among 413 children aged 6–17 years with asthma enrolled in a US trial of omalizumab, investigators found that overall nasal microbiota composition at baseline was not associated with exacerbation, but children who experienced exacerbations were more likely to possess assemblages dominated by *Moraxella* spp. and reduced *Corynebacterium* and *Staphylococcus* spp. (47). Interestingly, nasal microbiota composition remained stable during viral infections and exacerbations (47). Another study of asthma control examined nasal blow samples from 214 US children participating in a clinical trial of ICS; it found that children with nasal blow microbiota at baseline dominated by *Corynebacterium* and *Dolosigranulum* had lower rates of asthma exacerbation and longer time to asthma exacerbation compared with children with nasal blow clusters dominated by *Staphylococcus, Streptococcus*, and *Moraxella* (48). Also studying nasal blow samples, a separate study of 181 US children aged 6–17 years examined the effect of seasonality on the airway microbiome and childhood asthma exacerbation, finding *Moraxella* spp. and *Haemophilus* spp. to be associated with respiratory illness and asthma exacerbation in the fall; no significant associations between nasal microbiota and exacerbations were found in the winter, spring, or summer (49). A study of 344 Dutch children admitted with asthma exacerbations reported findings that were both consistent and contrasting to those of prior studies; compared with individuals acting as controls, the admitted children had high abundances of *Staphylococcus* and *Streptococcus* spp. but lower *Moraxella* spp. in their nasopharyngeal samples (50).

Taken together, airway microbiota associated with asthma control may differ by age, and while overlapping genera are reported across studies, the directions of effect are not always consistent. Multiple studies have reported associations between nasal *Prevotella* spp. and asthma exacerbations in adults, and many but not all studies of childhood asthma exacerbations have implicated imbalances in *Moraxella, Staphylococcus,* Streptococcus, and *Corynebacterium* spp. in upper airway samples. These findings have prompted in vitro experiments to assess the molecular effects of these species, including a study in which *Moraxella catarrhalis* was observed to induce more epithelial damage and inflammatory cytokine expression compared with other bacterial isolates (47). Additional experimental models can extend our understanding of how exacerbation-associated microbiota might functionally influence asthma control.

### Severe asthma.

Asthma severity is another dimension of asthma phenotyping shaped by symptoms, medication requirements, physiologic parameters such as lung function, and morbidity (51). Among a subset of adult participants in the European U-BIOPRED cohort, whole-genome sequencing of induced sputum showed that compared with 24 individuals without asthma acting as controls, α-diversity (Table 1) at the species level was lower, and there were increases in *Haemophilus influenzae, Moraxella catarrhalis*, and *Tropheryma whipplei* in the sputum of the 97 participants with severe asthma (29). Further study of U-BIOPRED participants with severe asthma revealed that participants with *Haemophilus influenzae* as the dominant species in induced sputum were more likely to have longer duration of disease, more daily oral corticosteroid (OCS) prescriptions, more exacerbations, and higher sputum neutrophil counts (52). Examinations of the airway microbiome associated with asthma severity in pediatric cohorts have yielded different findings. In a comparison of the nasal microbiome of 27 US children with severe persistent asthma versus 27 healthy children, investigators found significantly more abundant *Streptococcus* spp. in the nasal airway of children with severe persistent asthma without significant differences in α- or β-diversity (Table 1) (5). Overall, it is difficult to draw conclusions on airway microbiota associated with asthma severity given that study designs and findings have differed across cohorts and populations.

### Asthma with comorbid obesity.

Chronic inflammation underlies obesity and asthma, and given increasing rates of obesity, obesity-related asthma is a prevalent phenotype of asthma (53). To investigate the relationships among adult obesity, physician-diagnosed asthma, and the upper and lower airway and gastrointestinal microbiomes, investigators studied nasal, bronchoalveolar lavage (BAL), oral, and stool samples collected from individuals with obesity and asthma (*n* = 50), individuals without obesity and with asthma (*n* = 53), individuals with obesity and without asthma (*n* = 51), and individuals without obesity and asthma(*n* = 48) recruited from centers in Wroclaw, Poland, and Zurich, Switzerland (54). Relative to adults without obesity and asthma, those in the affected groups demonstrated variable pair-wise differences in the relative abundances of several bacterial genera in all sample types (54). In nasal samples, individuals with obesity and asthma had increased Shannon diversity, a measure of α-diversity (Table 1) (54). Another cross-sectional study of the airway microbiome in obesity and asthma examined induced sputum samples collected from 44 adults and 17 healthy controls from Michigan, USA (55). Regardless of asthma status, obesity was associated with different sputum bacterial community structure, including increased relative abundance of *Prevotella, Gemella,* and *Streptococcus* spp. and decreased relative abundance of *Leptotrichia, Campylobacter*, and *Centipeda* (55). Among participants with asthma, there was a body mass index–associated difference in sputum bacterial community structure after controlling for ICS use, although no difference in Faith’s phylogenetic diversity, a measure of β-diversity (Table 1) (55). Given that obesity contributes to systemic and airway inflammation (56), further research to uncover mechanisms by which airway microbiota might interact with obesity-induced inflammation to influence asthma outcomes would be of interest.

### Corticosteroid treatment.

Corticosteroids are a mainstay asthma treatment that also influence airway microbial communities due to their immunosuppressive effects and disruption of airway commensals (57). Research has been undertaken to examine the effects of corticosteroid treatment mode, dose, and treatment response on the airway microbiome. To examine the effects of corticosteroid treatment mode on the bronchial microbiome, investigators profiled endobronchial brushings and BAL fluid from 39 adults with asthma and 19 individuals acting as controls from the Chicago, USA, area (58). Compared with patients with asthma not on corticosteroids, the endobronchial brushings of those on ICS only or ICS and OCS had lower relative abundances of *Prevotella*, those on ICS and OCS had higher relative abundance of *Pseudomonas*, and those on ICS only had lower relative abundance *Veillonella* (58). In an examination of the effects of low- versus high-dose ICS treatment on the airway microbiome, researchers from the United Kingdom compared the sputum microbiome of 22 adults with asthma on low-dose ICS to that of 33 adults with asthma on high-dose ICS and found no significant differences in sputum bacterial load or overall community composition, although higher relative abundance of *Streptococcus* spp. was found in individuals taking low- versus high-dose ICS (59). With regards to ICS treatment response, a comparison of bronchial microbiota of US adult ICS responders (*n* = 10) to that of nonresponders (*n* = 5) found that bronchial brushings of ICS nonresponders were enriched in *Microbacteriaceae* and *Pasteurellaceae*, among other bacterial families, while responders’ bronchial brushings were enriched in *Streptococcaceae, Fusobacteriaceae*, and *Sphingomonadaceae* (60). Collectively, these studies demonstrate that administration route, dosage, and response to corticosteroids are all associated with airway microbiota. Understanding the degree to which airway microbiota causally influence corticosteroid response would be mechanistically informative and could help inform more effective asthma management.

### Asthma endotypes.

Asthma can be categorized into endotypes based on shared pathophysiologic mechanisms, with type 2–high asthma representing one prevalent and well-studied endotype characterized by elevated type 2 cytokines, eosinophil levels, IgE, and fraction exhaled nitric oxide (43). Type 2–low asthma, defined by the absence of type 2 inflammation and associated with treatment resistance, is less understood and may involve airway neutrophilia and elevated type 1 or Th17 inflammation (61). The success of biologic therapies targeting type 2 pathways underscores the clinical importance of endotyping asthma (62). Understanding the potential role of airway microbiota in asthma endotypes could advance endotyping efforts. Some studies of the airway microbiome in asthma have added to our understanding of established type 2–dependent endotypes, while other investigations have reinforced the complexity of endotyping and possibility of new ones.

Many studies have examined the airway microbiome associated with type 2–defined asthma, and here we describe a few examples. In a study of 84 US adults, including 42 individuals with atopic asthma, 21 individuals without atopic asthma, and 21 atopic healthy individuals acting as controls, investigators used the bronchial epithelial gene expression levels of 3 genes, *CLCA1, SERPINB2*, and *POSTN*, to calculate a type 2 score for asthma (60). They found that bronchial bacterial burden based on 16S rRNA copies negatively correlated with type 2 score across the participants with asthma and that participants with type 2–high asthma had lower bacterial burden than those with type 2–low asthma (60). Seeking to explore the fungal microbiome of asthma endotypes, other investigators profiled the fungal microbiome of BAL and endobronchial brushes obtained from 39 individuals with asthma and 19 individuals acting as controls from the Chicago, USA, area (63). They observed that relative to those with type 2–low asthma, participants with type 2–high asthma (defined based on peripheral blood eosinophil count) had lower fungal α-diversity in endobronchial brushes but not in BAL (63). The investigators also observed 12 exact sequence variants for fungi that differed between type 2–high and –low participants, including *Fusarium, Aspergillus, Cladosporium*, and *Alternaria* species in BAL and *Trichoderma* in endobronchial brushes (63). Another study of European individuals with asthma employed unsupervised methods that identified an airway microbiome cluster consistent with type 2–low asthma (64). Studying 100 adults with severe asthma and 24 adults with mild-to-moderate asthma from U-BIOPRED, unsupervised clustering of induced sputum microbiome profiles identified 2 main clusters, one of which was associated with blood and/or sputum neutrophilia, decreased sputum macrophages, and greater need for control of severe airway obstruction compared with the other cluster (64). This cluster had reduced relative abundances of a number of genera, including *Veillonella, Prevotella, Streptococcus, Rothia,* and *Haemophilus*, deficiencies thought by the investigators to contribute to increased risk of infection with pathogenic bacteria leading to the observed neutrophilia (64).

Other investigations of the airway microbiome and inflammatory patterns in asthma have demonstrated that some findings do not fall neatly into type 2–determined endotypes, highlighting the potential for alternative endotyping paradigms. In a study of BAL samples from 203 volunteers with asthma and healthy volunteers from the United Kingdom, researchers found that compared with participants with severe asthma with normal BAL IL-13 concentrations, participants with severe asthma with high BAL IL-13 despite steroid treatment had high BAL neutrophil percentages but not high BAL eosinophils (65). These individuals with severe asthma with high BAL IL-13 and BAL neutrophilia also exhibited correlated bacterial bronchial dysbiosis involving *Moraxella catarrhalis, Haemophilus sp*. and *Streptococcus sp*.(65). Additional studies have examined airway microbiota in conjunction with other airway molecular profiles to identify endotypes that overlap with but also go beyond those defined by type 2 status. A novel algorithm for jointly clustering clinical and multi-omic data via unsupervised methods was developed that when applied to an asthma cohort of 316 US individuals with clinical data and airway microbiome, transcriptome, and methylation profiles, identified 14 different clusters of asthma and health representing endotypes (66). While 2 endotypes corresponded to type 2 asthma, other endotypes defined by distinct molecular processes such as leukocyte and lymphocyte activation were also found by this integrative clinical and multi-omic algorithm (66). Of note, the identified endotypes of asthma differed in their relative abundances of nasal *Moraxella, Corynebacterium, Staphylococcus,* and *Streptococcus* (66). Also leveraging multi-omics, U-BIOPRED investigators jointly analyzed multi-omic data including 16S rRNA and metagenomics sequencing data from induced sputum collected from 57 patients with severe asthma, 15 patients with mild-to-moderate asthma, and 13 healthy volunteers (67). A clustering algorithm identified five omics-associated clusters, each representing variable levels of sputum neutrophilia and eosinophilia, asthma severity, atopy, smoking, cytokines, and dysbiosis, including one cluster marked by reduced α-diversity, high neutrophils, and enriched *Moraxella catarrhalis* and *Haemophilus influenzae* (67). The utility and generalizability of novel endotypes such as these will have to be tested in independent populations.

Given the value of endotypes to better target therapy, efforts to improve the endotyping of asthma remain of interest. Among many variables, airway microbiota could contribute to asthma endotyping, as the studies discussed in this section have shown that endotypes defined in various ways by airway microbiota are associated with clinical variables and outcomes. However, the efficacy of airway microbiota-defined endotypes will need to be demonstrated in prospective studies and across different populations before they will have practical impact in the clinical arena.

## Shaping the microbiome as asthma therapy

**Figure 4:**
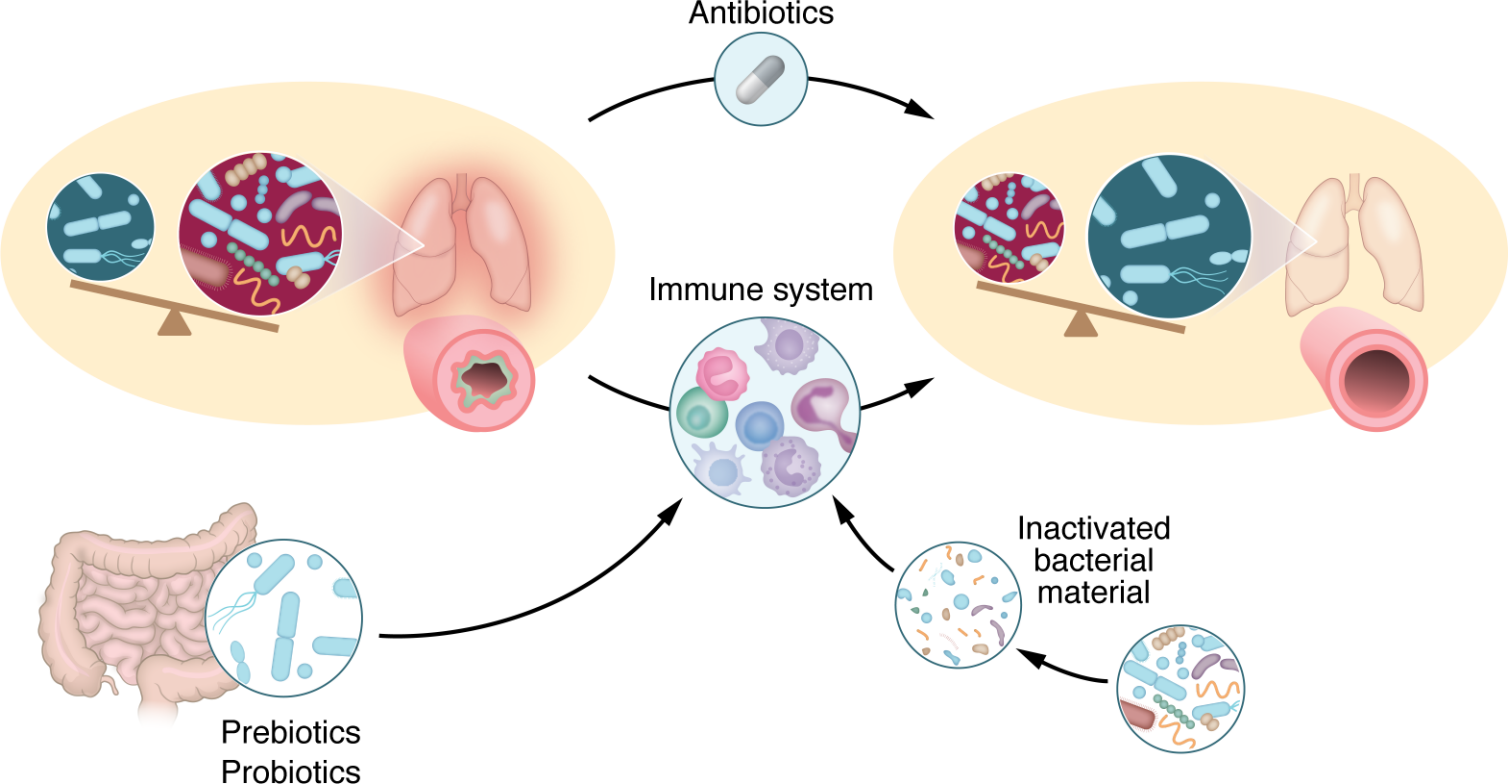
Microbial interventions are being explored as asthma therapy. Approaches that have been examined in human and murine studies include antibiotics, probiotic and prebiotic supplementation, and intake of inactivated bacterial material as immunotherapy.

As our understanding of the role of the microbiome in asthma deepens, interest also grows in trying to design interventions that might shape the microbiome for preventive or therapeutic benefit. Approaches examined in human studies thus far include orally delivered interventions, such as macrolide antibiotics (68), probiotic and prebiotic supplementation (69), and intake of inactivated bacterial material for immunotherapy (70) (Figure 4). While these interventions have greatest effects on the gut microbiome, the concept of the gut-lung axis suggests that intervention on the gut microbiome can influence the respiratory microbiome and airway immunologic pathways involved in the pathogenesis and disease course of asthma (71).

Given that the establishment of the early infant airway and gut microbiomes is linked to the maternal vaginal, skin, and gut microbiomes (72–74), oral probiotic supplementation for women during pregnancy and infants has been proposed for asthma prevention (75). Several randomized controlled studies of supplementation with oral probiotics to both pregnant mothers and infants, commonly with *Lactobacillus rhamnosus* GG (LGG), have not shown consistent benefit for childhood wheezing or asthma (76–80). While oral LGG supplementation of infants at risk for future asthma can partially and temporarily modify their gut microbiome, the effects are subtle and lost 6 months after cessation of supplementation (81). While conceptually compelling, the use oral probiotics during pregnancy or infancy for asthma prevention is not yet supported by the current literature.

Fewer investigations have focused on directly altering the airway microbiome for asthma treatment. A murine model study of allergic asthma examined intranasal administration of LGG, finding that it decreased BAL eosinophil counts, lung IL-13 and IL-5 levels, and airway hyperreactivity (82). Another murine study focused on aerosol inhalation of inactivated bacterial material (heat-killed *Clostridium butyricum*), showing that it reduced allergic airway inflammation and mucus secretion in allergic mice (83). It remains to be seen if these promising results from murine models can be translated into therapies that directly alter the airway microbiome in humans with asthma.

As microbiota likely exert their effects through metabolites they produce, there is interest in identifying microbial metabolites to leverage as therapy. Although the majority of studies to date have focused on gut-derived metabolites (84), interest in metabolites produced by airway microbiota is growing. Some studies have shown through metagenomic inference that airway microbiota associated with asthma and asthma control are involved in the metabolism of glycerolipids, short-chain fatty acids, and amino acids (45, 60). Glycerolipid signatures were also found in a metabolomic study of nasopharyngeal samples from infants with bronchiolitis, with complementary transcriptomics and drug signature mapping identifying potential therapeutic candidates such as prostaglandin modifiers for reducing disease severity (85). Further characterization of bioactive microbiome-derived metabolites in the airway, especially if findings are consistent across cohorts, could pave the way toward novel therapies.

While we do not yet have in hand an effective intervention to shape the airway microbiome toward enduring profiles of health and away from asthma, research along all fronts, from bench studies of the molecular effects of airway bacterial strains to clinical trials of live biotherapeutic products, are underway to inform the path ahead. With further research, especially with prospective designs and validation in cohorts with diverse representation, we anticipate that asthma therapy will one day include airway microbial interventions.

## Conclusions

Our studies of the airway microbiome in asthma have revealed a complex and dynamic ecosystem that plays an influential role in the development, progression, and management of asthma. Uncovering the intricate relationships and interactions between the airway microbiome and host immune system has and will continue to offer new insights into asthma pathogenesis and potential therapeutic targets. However, many questions remain unanswered. While several bacterial genera have been associated with risk of asthma development and asthma morbidity, finer resolution work to identify the most impactful species, strains, and communities, including their causal effects on immune pathways and molecular networks, is needed to advance mechanistic understanding and facilitate translation into potential therapies. Future research will also benefit from longitudinal designs to better characterize the temporal dynamics of the airway microbiome in asthma, the functional implications of microbial-host interactions, and the development of microbial-based diagnostic and therapeutic strategies. Diversifying the geographical scope of airway microbiome studies beyond the United States and Europe will also enable better capture of global variation in exposures, the airway microbiome, and asthma, thereby expanding the relevance and impact of findings (16, 86, 87). The effects of climate change on the airway microbiome and asthma risk will also need to be increasingly considered. Additionally, as we move toward more personalized approaches in asthma management, integrating microbiome data with clinical, molecular, and environmental information can lead to more effective prevention strategies and targeted treatments. While challenges remain in translating microbiome research into clinical practice, the field holds promise for improving outcomes for individuals with asthma.

## Figures and Tables

**Table 1:**
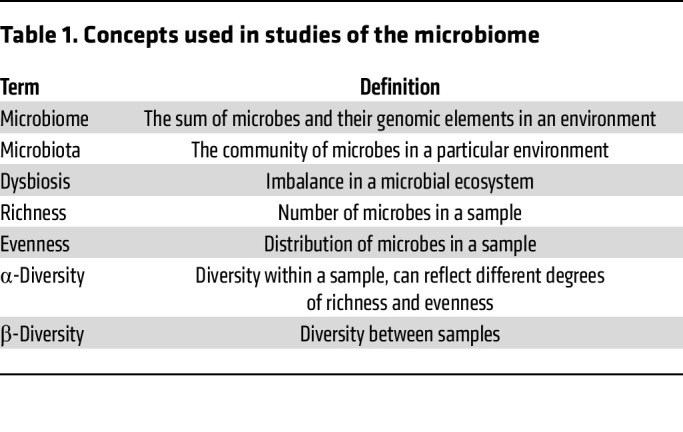
Concepts used in studies of the microbiome

## Version 1 02/17/2025

Electronic publication
